# Detection of exercising ectopic atrial and ventricular beats using non-linear analysis of clinically normal racehorse electrocardiograms at rest or low-intensity exercise

**DOI:** 10.1038/s41598-026-41281-0

**Published:** 2026-03-13

**Authors:** Vadim Alexeenko, Hamid Tavanaeimanesh, Freya Stein, Jenifer Gold, Lauren Hughes, Molly McCue, Celia Marr, Sian Durward-Akhurst, Kamalan Jeevaratnam

**Affiliations:** 1 University of Surrey, Guildford, UK; 2 Department of Veterinary Clinical Sciences, University of Minnesota, Minnesota, USA; 3 Wisconsin Equine Clinic, Oconomowoc, Wisconsin USA; 4 Department of Veterinary Population Medicine, University of Minnesota, Minnesota, USA; 5 Rossdales Veterinary Surgeons, Newmarket, UK

**Keywords:** ECG, Horse, Complexity, Performance, Cardiovascular diagnostics, Algorithm, Predictive medicine, Arrhythmias

## Abstract

Cardiac arrhythmias are common in healthy athletic horses and may lead to poor athletic performance or exercise-associated sudden death. Early detection of high-risk horses is an important goal of cardiovascular diagnostics. We hypothesised that non-linear analysis of electrocardiogram disorderliness can be used to identify horses exhibiting intermittent ectopic atrial and ventricular heart rhythm abnormalities at exercise using brief, artifact-free recordings of normal sinus rhythm electrocardiograms collected at submaximal heart rates. In a convenience prospective cross-sectional study, ambulatory electrocardiograms were recorded using the Televet 100 or II devices from 110 Thoroughbred or Standardbred racehorses during routine training. Acceptable quality 60 s electrocardiogram strips with stable heart rate (20–120 beats per minute) were identified automatically. Disorderliness of the electrocardiograms was estimated using Lempel–Ziv’76 and ’78, and Titchener complexity, and Shannon, sample, and approximate entropy algorithms. Numerical estimates obtained by these algorithms were corrected to the heart rate. For the optimal performance recordings of 60–100 beats per minute should be used, with Lempel–Ziv ’76 complexity, R peak and ends of S and T peaks as fiducial points. The receiver operating curve analysis has demonstrated the area under curve of 0.86 for this combination, indicating acceptable differentiation between cases and controls.

**Supplementary Information:**

The online version contains supplementary material available at 10.1038/s41598-026-41281-0.

## Introduction

Intermittent irregularities of normal heart rhythm, including ectopic beats and cardiac arrhythmias, are extremely common in athletic horses. Up to 50% of healthy Standardbred^1^ and 92% of healthy Thoroughbred^2^ racehorses have at least one premature complex during or immediately following exercise. The clinical consequences of these arrhythmias range from no apparent effect to poor athletic performance^3^. Severe cases of exercising arrhythmias are thought to contribute to exercise associated sudden death (EASD) (approximately 10% of total causes of racing mortality)^4^. One of the main challenges to determining the significance and consequences of these arrhythmias is identifying horses that develop them for prospective long-term monitoring of high-risk horses.

The clinically accepted method for exercising arrhythmia diagnosis in horses relies on exercising electrocardiograms (ECGs), which are more laborious to obtain and typically contain significant movement artefacts. Interpretation of these ECGs is a challenge, frequently requiring a specialist; therefore they are not routinely performed, except for during cardiac evaluation of poor performance. In comparison, resting or low-intensity exercise ECGs are easy to obtain and interpret due to reduced movement artefacts compared to maximal intensity exercising ECGs.

We have shown that paroxysmal atrial fibrillation (PAF), which is the most frequent sustained arrythmia in both horses and humans, is linked to alterations in normal sinus rhythm ECGs, even in ECGs that would be classified as clinically normal by expert clinicians. These inconspicuous alterations can be identified using non-linear analyses including ECG disorderliness assessment by complexity estimators^5,6^, and ECG restitution^7^. Complexity analyses estimate the disorderliness of the inherently chaotic activity of the heart^8,9^. It could be expected that the pathophysiological changes in the myocardium that increase risk of arrhythmia development would alter the patterns of wavefront travel across the heart. Similarly, the ability of the heart to recuperate after the beat would be affected by pro-arrhythmic alterations which include fibrosis, channelopathies and other biochemical changes. Hence, ECG restitution analysis, based on comparison of various interval durations within the heartbeat, may also be an efficient tool for early arrhythmia detection^10,11^.

Both complexity and restitution analyses can be performed using artefact-free short ECG strips (30–60 s) recorded in normal sinus rhythm at heart rates less than 100 bpm. Such ECGs can easily be recorded at submaximal intensity exercise. At these heart rates automatic annotation is simpler due to reduced movement artefacts and clear separation of individual peaks within the heartbeat waveform, which is not true for maximal intensity exercising ECGs. The optimal sampling frequency for human ECG disorderliness analysis is close to 125 Hz, which is easily attainable using commercially available ECG recording devices^12^. While differences in electrode placement and signal processing settings of various ECG devices affect the signal shape, appropriate corrections can be derived to ensure interoperability between devices.

**Fig. 1:**
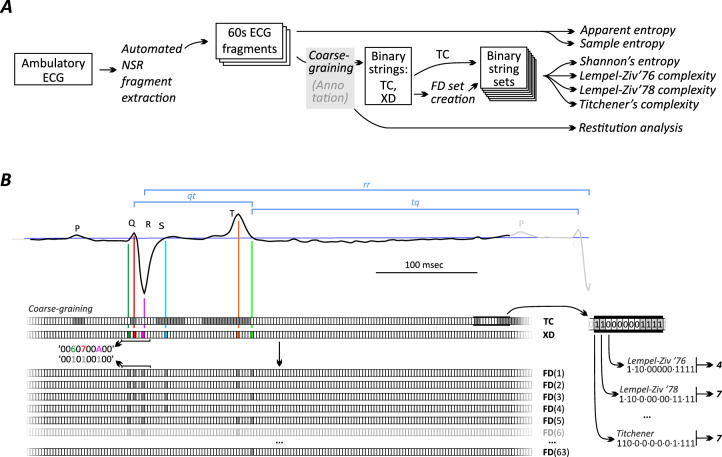
ECG analysis pathway. (**A**) ECG processing pipeline. ECG coarse graining for complexity analysis and annotation for restitution analysis use the same algorithm. (**B**) ECG coarse graining/annotation and complexity analysis technique. *NSR* normal sinus rhythm, *TC* threshold crossing, *XD* extended feature detection, *FD* feature detection. XD string is used to generate the set of 63 FD strings. Blue lines show intervals used for ECG restitution analysis. Enlarged portion of TC binary string demonstrates difference in parsing techniques among several complexity estimators.

ECG disorderliness estimation methods can be broadly separated to those that require prior conversion of ECG waveforms to strings of symbols, including Shannon entropy, Lempel–Ziv ’76 and ‘78, and Titchener complexities, and those that process the raw voltage signal, including sample and approximate entropy (Fig. 1). The first group of estimators decomposes the source string of symbols to a set of substrings needed to rebuild the original symbolic string by simple copy and extend algorithms. The number of substrings generated by the decomposition algorithm is used as a metric of signal complexity. The second group of estimators looks for repetitions of patterns in the original signal without the need for the signal conversion step. For detailed description of complexity estimators the reader is referred to the original works of Lempel and Ziv^13^ and Titchener^14^ complexity estimators, and Shannon^15^, approximate^16^ and sample^17^ entropies. Due to the non-linear nature of all these methods, any alterations in the signal pre-processing parameters and choice of estimator may affect the diagnostic method performance. This requires a thorough scrutiny of all possible combinations of analysis and pre-processing parameters to develop the best possible method for differentiating between subjects with and without arrhythmias.

Pre-processing of the ECG may hide or highlight features of the ECG waveform associated with distinct periods of electrical activity of atria or ventricles. To detect supraventricular and ventricular rhythm abnormalities it may seem logical to focus exclusively on features linked with atria and ventricles correspondingly. However, the pathophysiological alterations causing rhythm disturbances are not necessarily confined to the tissue of the chamber where the disturbance originates^18^. Therefore, our study was set to systematically examine the contribution of all studied ECG features for detecting both atrial and ventricular ectopic beats at exercise, based on the assumption that changes in the myocardium extend beyond ectopic foci. The analysed set of features includes Q, R, and T peaks, onset of Q peak, termination points of QRS complex and T peak. Within the set, all features were sequentially and systematically excluded in all possible combinations, thus previously-used feature-detection (FD) and beat-detection (BD) strings were analysed as parts of a larger FD set of strings.

ECG restitution technique relies on the intervals of heart contraction and relaxation within a heartbeat being altered by pathophysiological changes that develop due to arrhythmia presence. Arbitrary formulae can be devised for quantification of these relationships, however interpretation of ECG restitution data can be feasibly performed by machine learning algorithms operating in multidimensional space, for example, k-NN classifier using triplets of TQ, QT and RR intervals^7^.

Our hypothesis is that using a combination of ECG features and complexity estimators or restitution analyses of normal sinus rhythm ECGs recorded pre-exercise at submaximal heart rates, racehorses that develop ventricular and atrial ectopic beats at exercise can be differentiated from those without ectopic beats at exercise. These potentially high-risk horses can then be monitored more closely to determine what, if any, clinical consequences develop in these horses, including worsening arrhythmia severity, poor performance and/or EASD.

## Results

### ECG complexity dependence on the heart rate is affected by features selected for analysis

**Fig. 2:**
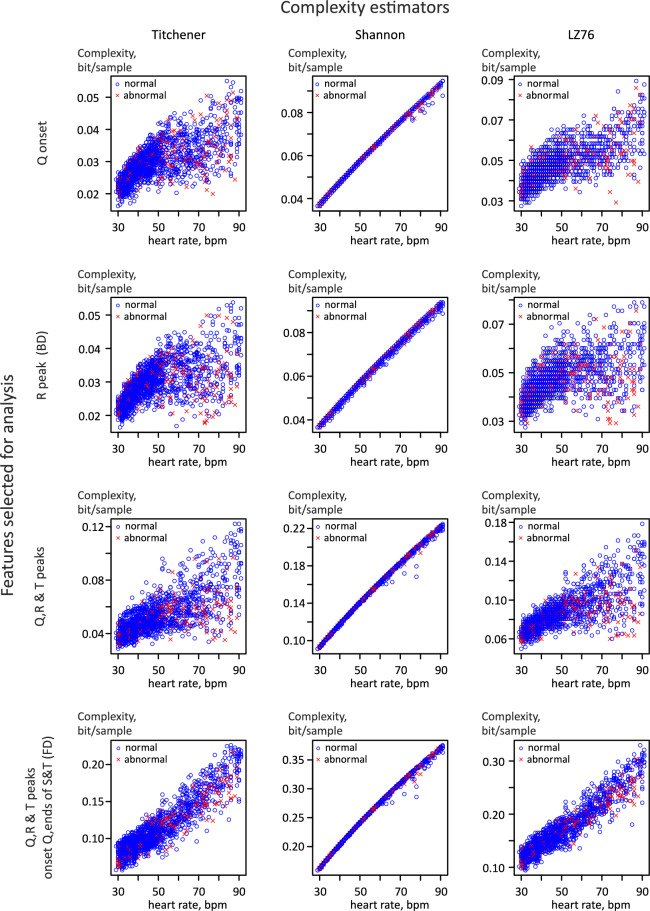
Dependence of ECG complexity on heart rate for different complexity estimators. Different complexity estimators are shown in columns and different feature sets in rows.

To evaluate the dependency of complexity values over heart rate in different combinations of ECG preprocessing techniques and complexity estimators we plotted these dependencies for all available combinations of markers and complexity estimators. An abbreviated sample of plots is shown in (Fig. 2). We observed that the dependency of complexity values on heart rate is non-linear and highly dependent on the combination of markers and complexity estimators. Therefore, to perform a robust detection algorithm would require appropriate correction for the heart rate as described in the materials and methods. Correction of the heart rate was performed for all individual combinations of complexity estimators and methods of ECG strip preprocessing.

### Optimisation of heart rate limits for detection of ectopic beats

**Fig. 3:**
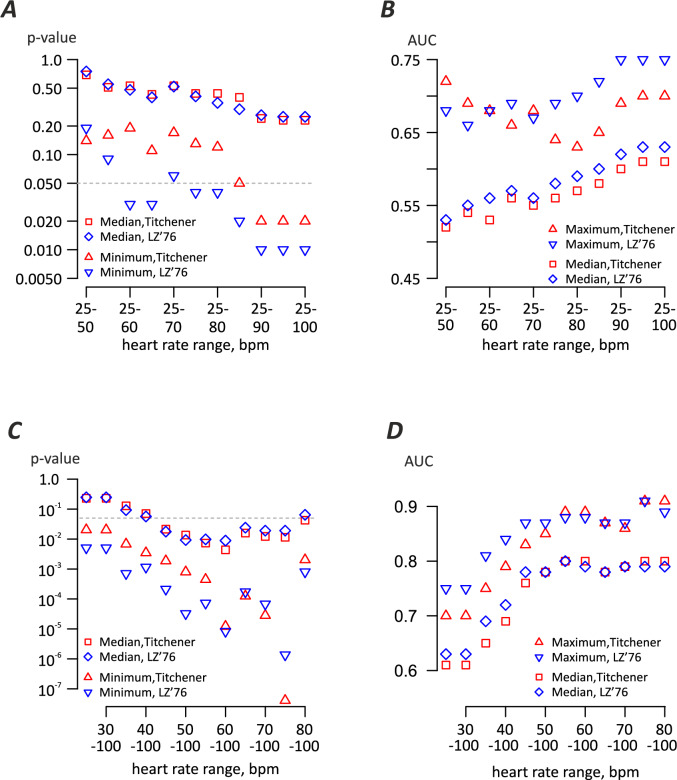
Analysis of optimal heart rate ranges for the detection of ectopic beats from normal sinus rhythm ECGs. (**A**) p-value of the difference between heart rate corrected complexity between cases and controls, with upper heart rate limit varied. Median p-value and minimum p-value across 63 FD parsing schemes for Titchener and LZ’76 complexity estimators shown. Dashed line is at significance level of 0.05. (**B**) Receiver operating curve dependence on heart rate range analysis for the data shown in panel *A*. Maximum AUC and median AUC values are shown. (**C**) Analysis of lower bound selection influence, using p-value as metric. Dashed line is at significance level of 0.05. (**D**) Receiver operating curve dependence on heart rate range analysis for the data shown in panel *C*. Note that analysis of data in heart rate range above 80 bpm is important for high discrimination performance (**A**,**B**) while analysis of data at heart rates below 60 bpm is detrimental for it (**C**,**D**).

To explore the impact of heart rate on the performance detection algorithm, we analysed the influence of upper and lower bound of heart rates on differentiation between controls and cases. In an initial study we fixed the lower heart rate limit at 25 beats per minute (bpm) and varied the upper heart rate limit from 50 to 90 bpm. The median and minimum p-value for the difference between two cohorts across 63 marker sets was used to quantify the algorithm performance (Fig. 3A). Additionally, we calculated the area under the curve (AUC) values for receiver operating curve analysis for the same combinations (Fig. 3B). The results of analysis clearly indicate that optimal performance requires analysis of ECGs recorded at submaximal exercise heart rates, with top limit in 80–100 bpm range. The median and minimum p values decreased for both LZ’76- and Titchener-based algorithms when the maximum heart rate was 90 – 100 bpm and the lower heart rate was 25 bpm, while median and maximum AUC values increased (Fig. 3A, B). Similarly, when fixing the upper heart rate at 100 bpm and varying the lower heart rate, lower heart rates below 60 bpm had the highest p values and lowest AUCs for LZ’76- and Titchener-based algorithms (Fig. 3C,D).

The algorithms based on apparent entropy, sample entropy, Shannon entropy and LZ’78 complexity were unable to efficiently discriminate between cases and controls groups in conjunction with any source strings and heart rate ranges and were therefore excluded from further consideration.

### Characterisation of best performing algorithms

**Fig. 4:**
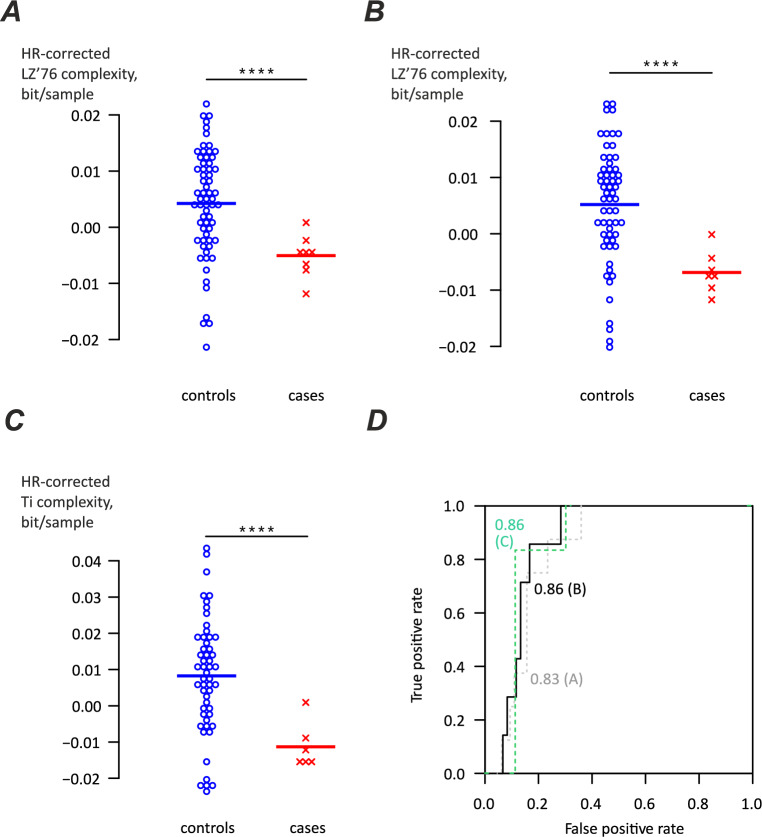
Performance of complexity algorithms for different heart rates, for combinations of complexity estimators and markers producing lowest p values in comparison of cases and controls. (**A**) Heart rates 50–100 bpm, LZ76 complexity estimator, using markers of T peak and ends of S and T peaks. (**B**) Heart rates 60–100 bpm, Titchener complexity estimator using markers for Q, R, T peaks and ends of S and T peaks. (**C**) Heart rates 70–100 bpm, Titchener complexity using markers for Q and T peaks and ends of S and T peaks. (**D**) Receiver operating curve analysis, for panels A-C. Area under curve values are shown by the curve together with the panel index in parentheses: grey dotted line for panel A, black line for panel B, green dashed line for panel C.

The above algorithm performance analysis allowed us to narrow down the selection of best performing algorithms to ones using Lempel–Ziv’76 and Titchener complexity over a heart rate range of 60–100 bpm (Fig. 4B). The best performing algorithms were selected based on markers allowing to obtain lowest p-values for the difference between cases and controls even if ROC analysis produced higher AUC values for some other marker set.

The best performing algorithm for the heart rate range 60–100 bpm used LZ’ 76 complexity and ECG features including R peak and ends of S and T peaks (Fig. 4B). The heart-rate corrected complexity for the controls was significantly higher (p < 0.001) at 0.00519 ± 0.0103 bit/sample (n = 60) compared to cases − 0.00686 ± 0.00389 (n = 7). The AUC was of 0.86 (black solid line, Fig. 4D). For comparison, the best performing algorithm at 50–100 bpm, employed LZ’76 complexity, T peak and ends of S and T peaks (Fig. 4A), and the complexity for the controls was 0.00424 ± 0.00958 bit/sample (n = 64) and for cases − 0.00506 ± 0.00373 (n = 8), with p < 0.001 and area under curve of 0.83. The increase of lower HR limit to 70 bpm provided little improvement as with the algorithm using Titchener complexity, Q, R and T peaks and ends of S and T peaks (Fig. 4C), the complexity for the controls was 0.00825 ± 0.0159 bit/sample (n = 53) and for cases − 0.0113 ± 0.00606 (n = 6), with p < 0.001 and area under curve stayed at 0.86.

Out of 16 combinations of parsing schemes and complexity estimator providing the highest AUC (exceeding 0.85) for the ECGs in 60–100 bpm range, 11 used Titchener complexity and only five LZ’76; the most frequently encountered feature was the end of QRS complex (15 occasions), followed by T peak (11 occasions), Q peak and end of T wave (9 each), and R peak (8).

The calculation of estimates of algorithm performance for the algorithm using ECGs at 60–100 bpm range, LZ’76 complexity, R peaks and ends of S and T peaks returned sensitivity of 85.7% (confidence interval 59.8–100%), specificity 83.3% (confidence interval 73.9%-92.8%), positive predictive value of 37.5% (confidence interval 13.8- 61.2%) and negative predictive value of 98.0% (confidence interval 94.2–100%).

### ECG restitution analysis

To explore the usability of ECG restitution analysis we used the same 60 s ECG segments as were used for the complexity analysis. The exploratory analysis suggested that the data belonging to the cases and control groups are overlapping and any analysis lacked sufficient power to produce efficient differentiation between groups.

## Discussion

We demonstrated that horses with supraventricular and ventricular ectopic beats at exercise can be identified using disorderliness analysis of normal sinus rhythm pre-exercise and at submaximal intensity exercise ECGs using complexity estimators and techniques conceptually similar to ones which were shown to be applicable for detection of paroxysmal atrial fibrillation (PAF) in horses. However, there are some notable differences in data preprocessing and heart rate range required to obtain maximum sensitivity of this method for identifying exercising ectopics in comparison to the methods used for horses with PAF. At this time, restitution analysis does not appear to be useful for differentiating between horses with exercising ectopic beats and those without, due to extensive overlap of interval duration data in control and case cohorts.

**Fig. 5:**
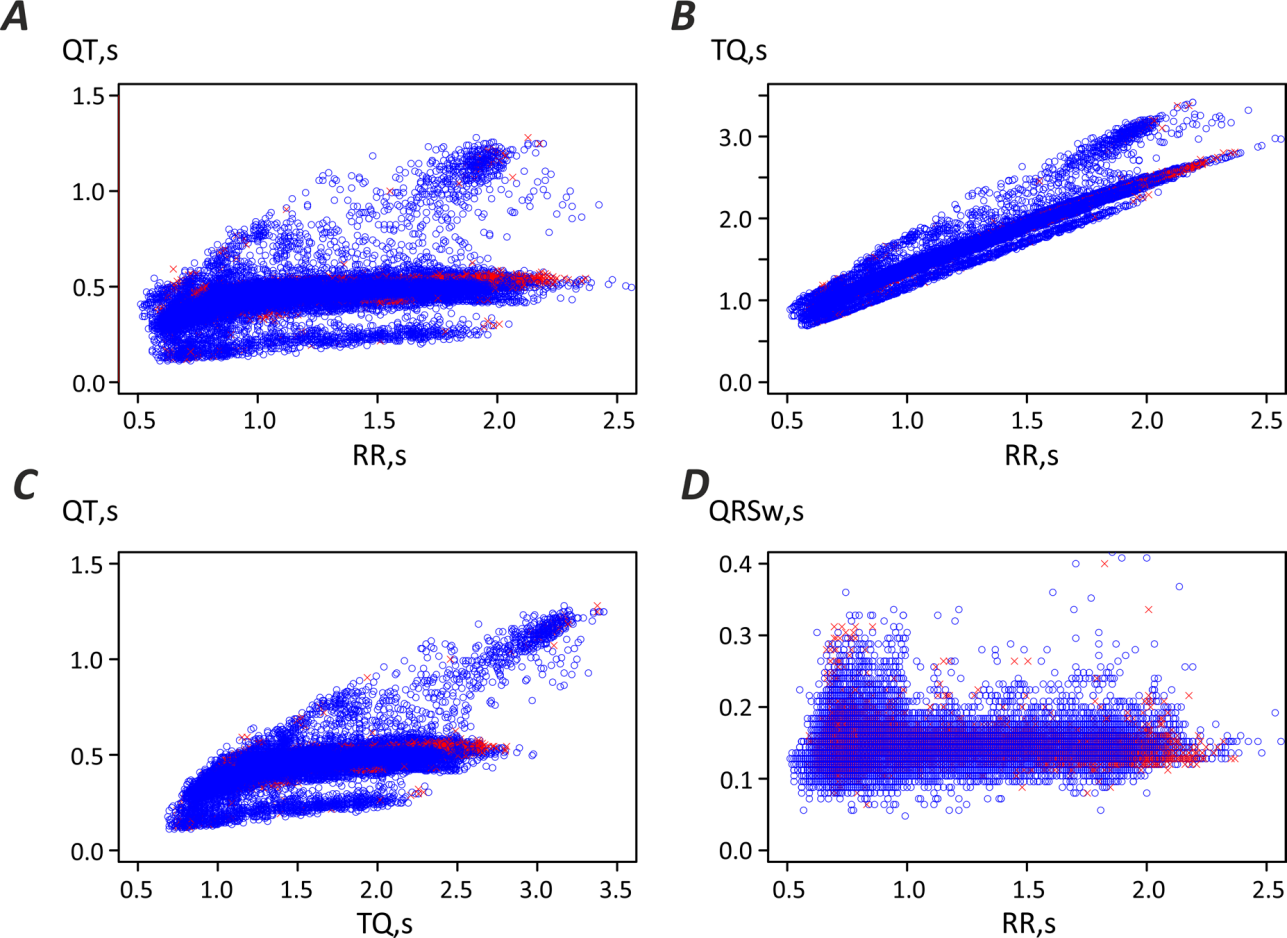
Restitution analysis. No distinct difference was observed for control (blue) compared to case (red) ECG segments. (**A**) Dependence of the action potential duration (represented by QT interval) on the basic cycle length (RR interval). (**B**) Dependence of the diastolic interval (represented by TQ interval) on the basic cycle length (RR). (**C**) Dependence of the action potential duration (QT) on the diastolic interval (TQ). (**D**) Dependence of the QRS complex width on the basic cycle length (RR).

The observation that while PAF diagnostics requires the analysis of ECGs recorded below 60 bpm and ectopic beat detection at heart rates above 60 bpm is remarkable. First, it has the potential to allow for simultaneous checking the patient for various heart rhythm abnormalities within a single diagnostic session, recorded at rest and before high intensity exercise, which corresponds to a walk or trot—and should convey low risk of triggering peak exercise intensity associated arrythmias. It also raises the important question about the nature of such difference. We suggest that it may be linked to the adaptation of the heart as the sympathetic tone increases as the horse prepares for higher intensity exercise as opposed to rest. The ECG restitution analysis demonstrates (Fig. 5) that above 60 bpm (below an RR interval of 1 s) dependence of QT interval on RR interval is different than at lower heart rates, which is consistent with previous reports^19^. The same is true for dependence of QT and TQ intervals.

We explored the usability of several complexity estimators across different heart rate ranges and using different coarse graining schemes which retained information of different features of the ECG waveform. Our observation that optimal performance of the best complexity-based algorithm requires analysing the signals from the T-wave which corresponds to repolarisation of the heart after the beat. This could suggest that cardiac repolarization is altered in horses with ectopic beats at exercise. Causes of altered cardiac repolarization include variation in ion channels, altered autonomic nervous system balance, or alterations in the myocardium that can develop in individuals with repeated arrhythmias. The QT interval is known to vary in people with certain arrhythmic syndromes and has been shown to be a predictor of mortality^20^. To date, no association between the QT interval and equine mortality has been reported.

We further demonstrated that Titchener’s complexity estimator might be an important tool for the analysis of biological signals, most likely due to its slightly better performance at the analysis of short strings^21^. It was previously shown that optimal sampling frequency for PAF detection is 125 Hz^12^ and it might be expected that the same would hold true for ectopic beat detection. This means that with the heuristic practical limit of equine ECG signal duration of one minute or so, only 7500 samples would be analysed. While this length of a signal is longer than ‘very short’ signal of 2000 or less samples^22,23^, it still may be conveniently classified as a ‘short’ signal^21^. Our conclusions agree with Speidel’s suggestion^21^ that ‘sensitivity of T*(Titchener’s)*-complexity is higher, suggesting that it may be more sensitive while suffering less from estimation noise’. However, performance of a simpler algorithm (LZ’76) appears to be very close to Titchener’s, and thus a dedicated comparison of precision of both algorithms for ECG analysis may be required.

In the last decade numerous attempts were done to use artificial intelligence-based classifiers to predict arrythmias from normal sinus rhythm ECGs. While very successful, these approaches do not determine the physiological basis for such classification. Complexity-based methods have a mechanistic explanation of assessing the non-uniform alterations in conduction in the excitation wavefront travel through the heart. It might be interesting to compare the performance of brute force artificial intelligence algorithms and complexity-based algorithms on the same data.

Similar to our findings in PAF, complexity in horses with ectopic supraventricular or ventricular arrhythmias at exercise is lower than horses without ectopic beats at exercise. This may be related to cardiac remodelling due to repeated exercising arrhythmia presence. This study however was a cross-sectional study, and we do not have repeated ECGs for a large proportion of the horses included here. The best performing complexity algorithms for exercising ectopic beats used Titchener or Lempel–Ziv ’76 complexity, and end of QRS complex and T peak. This would suggest that both ventricular depolarisation and repolarisation are altered in horses with ectopic beats at exercise. The clinical significance of this is not clear as alterations are subtle, but warrants further investigation of the best resting or pre-exercise ECG markers for previous PAF and ectopic beat at exercise detection.

The accuracy of our test indicates that while sensitivity and specificity of the test might be high, close to 0.9, and negative predictive value close to 100%, positive predictive value is low – only around 0.4. Despite this being relatively low, it is comparable to the AUC of 0.4 for a method that is currently used for PAF detection using a multi- day Holter ECG recording in humans^24^. Given the short duration of resting or pre-exercise ECG required and the excellent negative predictive value, this makes this method a potentially excellent screening test for ectopic exercise arrhythmia detection. Horses that screen positive based on this test could be marked to undergo an exercising ECG to determine the type, severity, and frequency of arrhythmia that is present at exercise.

There are several limitations for this study. First, it was reliant on convenience sampling; therefore, data analysis was constrained by the availability of ECG data in the heart rate range of interest. Second, our algorithm for correction of complexity values for the heart rate uses the ad hoc algorithm which is based on the data collected for this study. Such approach might be appropriate for a pilot study, however a deeper analytical study using larger samples would be beneficial to derive the algorithms appropriate for standardisation of the method. Finally, the cases and controls were classified based on only a single ECG, given that some horses may have normal ECGs during one workout and arrhythmias on another, there may be some overlap between cases and controls at different time points. This could significantly reduce the estimates of algorithm performance.

## Conclusion

Our study demonstrates that disorderliness analyses of the normal sinus rhythm ECG can be used for detection of ectopic beats that occur during exercise while analysing normal sinus rhythm pre maximal intensity exercise ECGs. The method we suggest requires only submaximal intensity exercise and can be performed in conjunction and using the same techniques as a technique we suggested for PAF detection previously. This study demonstrates the feasibility of non-linear analysis as predictors of cardiovascular changes which may be used in conjunction with AI algorithms to decrease exercise associated mortality in horses. There is also potential translational potential as these results are consistent with our previously demonstrated PAF discovery methods that have been used in detection of human PAF^5^.

## Materials and methods

### Horse ECG examination

This study was approved by the University of Minnesota Institutional Animal Care and Use Committee, protocol number 2208-40347A. All experimental procedures in the study were non-invasive and were performed in accordance with relevant guidelines and regulations. Using a prospective convenience sampling method, we performed cardiac examinations on 110 Standardbred and Thoroughbred racehorses that were in active race training. Cardiac examinations include cardiac auscultation and placement of an ECG (Televet 100 or II, Engel Engineering) before, during, and immediately after exercise. Exercise intensity was determined by the trainer and we tried to only sample horses doing high intensity exercise. Horses with potentially clinically relevant heart murmurs (grade $\ge$ 3/6) were excluded from the study. Electrode placement followed a modified base-apex configuration (Supplementary Figure S1). Two internal medicine-boarded and one internal medicine-trained specialist reviewed the ECGs (SDA, FS, and HT). All horses had to have at least one minute of resting ECG with minimal artifact. Case horses had at least five premature depolarizations and/or at least one episode of complexity, including couplets, triplets, and/or supraventricular or ventricular tachycardia during high intensity exercise (heart rate > 180 beats per minute) or immediately after exercise (during the period of rapid deceleration which is approximately two minutes post peak intensity exercise). Inclusion criteria for control horses was a complete exercise and immediate post exercise recovery period ECG, no episodes of complexity, and having less than five premature depolarizations. Horses with atrial fibrillation observed in the ECG or a history of atrial fibrillation were excluded. The total number of horses available for the study was 110. Some horses were excluded due to the automated ECG extraction algorithm determining they did not have acceptable quality ECG recordings. The final data set included 90 subjects (47 geldings, 40 mares, 3 stallions, 65 Thoroughbreds and 25 Standardbreds), average age of 4.2 ± 1.8 years. Of these, control cohort included 80 subjects (age 4.2 ± 1.9 years, 42 geldings, 35 mares, and 3 stallions; 60 Thoroughbreds and 20 Standardbreds), and cases cohort included 10 subjects (age 4.3 ± 1.3 years, 5 geldings and 5 mares; 5 Thoroughbreds and 5 Standardbreds). In a typical training session heart rates typically stay below 120 bpm for majority of the time, and higher heart rates are observed for < 20 min (see Supplementary Figure S2).

### ECG segment extraction

The original ECG data files were exported to text-only format (comma-separated values, CSV) files using Televet software. To eliminate human selection bias, the *FIND_SEGMENT* tool was developed by the authors using C +  + language. This tool detects R peaks and analyses R-R intervals as a criteria of ECG segment usability. Strips demonstrating beats with R-R intervals deviating more than 30% from the average R-R intervals were excluded, as well as strips with heart rate outside the pre-defined heart rate range as described below. The 30% threshold was selected empirically and is sufficient to exclude bradyarrhythmias such as second-degree atrioventricular or sinus block and sinus pauses as well as artifacts or disconnection, but will not exclude respiratory sinus arrhythmia. Non-overlapping ECG strips of suitable quality were then selected for further analysis. Baseline wander in these strips was eliminated by fitting a polynomial spline at the middle of R-R intervals, and the resulting polynomial curve was subtracted from the original ECG. All strips were down-sampled to 125 Hz sampling frequency, which was used in our previous studies^6,7,12^.

For complexity analysis, 60 s artefact-free ECG segments (strips) with stable heart rate between 30 and 120 bpm were extracted. This heart rate range was selected as previous studies demonstrated the feasibility of detection of alterations associated with paroxysmal atrial fibrillation in normal sinus rhythm ECG recordings at this heart rate range^6,7^. The final analysed data set included 307 60 s ECG strips from 67 horses. The median (interquartile range) heart rate was 73 (65–82) beats per minute. Each horse had a median (interquartile range) of 3 (1–7) resting ECG strips.

### ECG preprocessing

Shannon, Lempel–Ziv, and Titchener complexity estimators require ECG preprocessing. The ECG signal was coarse-grained (converted to text strings) using methods of Threshold Crossing (TC) and Feature Detection (FD). The TC method replaces the readings below the ECG median voltage of the analysed strip by ‘0’ and the rest by ‘1’. The FD method is aimed at investigating the role of individual ECG fiducial points within the heartbeat and FD strings were generated using intermediate step via eXtended feature Detection (XD) strings. XD coarse graining scheme uses the same annotation algorithm as previously described FD method, however, it replaces the ECG voltage value by ‘6’ at the onset and by ‘7’ at the peak of the Q wave, by ‘A’ at the R peak, ‘G’ at the termination point of the QRS complex, and ‘J’ and ‘L’ at the peak and termination of the R wave; all remaining values are replaced by ‘0’. Then, a set of FD strings is generated by replacing non- ‘0’ characters in the XD string with ‘1’, if the corresponding feature was included in the analysis, and by replacing non-converted characters to ‘0’. The final FD string set for the six assessed markers consisted of 63 (2^6^–1) FD strings. Apparent and sample entropy estimators do not require any additional pre-processing.

Apparent and sample entropy estimators did not require any additional pre-processing and were used as extracted.

### Disorderliness estimation algorithms

Estimation of symbolic string disorderliness was performed by a custom implementation of appropriate algorithms in C +  + language, using a Linux operating system and Qt framework (http://www.qt.io). This program simultaneously performed complexity analysis using several previously described methods: Lempel–Ziv ’76^13^, Lempel–Ziv ’78^25^ and Titchener T-complexity^14^, as well as Shannon, apparent and sample Entropy. Detailed descriptions of these decomposition methods can be found in corresponding publications. Briefly, the three methods estimate the disorderliness of symbolic strings by identifying the number of sub-strings (factors) required to build the source string according to the rules set by the authors of individual algorithm.

### Correction of complexity values for the heart rates and calculation of final metrics

To eliminate the dependency of complexity values on the length of the source string (n), LZ 76 complexity values were normalised to the n/log_2_(n) value^26^ and LZ 78 values were normalised to sequence length. For that purpose, optimal 2nd to 4th degree polynomials were fitted for averaged complexity data values using Akaike’s ‘An Information Criterion’ R tool. The resulting fitted curve was subtracted from the individual data sets. The resulting complexity values were not dependent on heart rate and used for further analysis. The average heart rate corrected complexity value for the subject’s data set was used as a metric for differentiation cases and controls. For the T-complexity, average entropy values were used. For the Shannon, approximate and sample entropy the output of the corresponding algorithm was used directly without normalization.

### Statistical analyses

Parametric data are expressed as mean ± standard deviation of mean. Demographics data was calculated using Microsoft Excel. Statistical analyses and plotting were done using GNU R 4.4.1^27^. A two-sided t-test (using Welch’s correction for unequal variances) was used for two-group comparisons with significance between data sets accepted at p < 0.05. The diagnostic performance of the method (sensitivity, specificity, negative and positive predictive values, and clinical utility index) was calculated using the Clinical Utility Index calculator created by AJ Mitchell^28^.

## Supplementary Information

Supplementary Information.

## Data Availability

Raw data and software used in the study are available from the corresponding authors upon the reasonable request and subject to signing the appropriate non-disclosure and material transfer agreements.
