# Supplementary material for: Detection of exercising ectopic atrial and ventricular beats using non-linear analysis of clinically normal racehorse electrocardiograms at rest or low-intensity exercise
Source: Sci Rep. 2026 Mar 13;16:13357. doi: 10.1038/s41598-026-41281-0 (PMC13106846; doi:10.1038/s41598-026-41281-0)

Supplementary Information for the submission ID: b8b60156-2950-4982-bd1c-91907aab83b0,

“Detection of exercising ectopic atrial and ventricular beats using non-linear analysis of clinically normal racehorse electrocardiograms at rest or low-intensity exercise”.

# Supplementary figures

**Figure S1** Electrode placement on the horse.

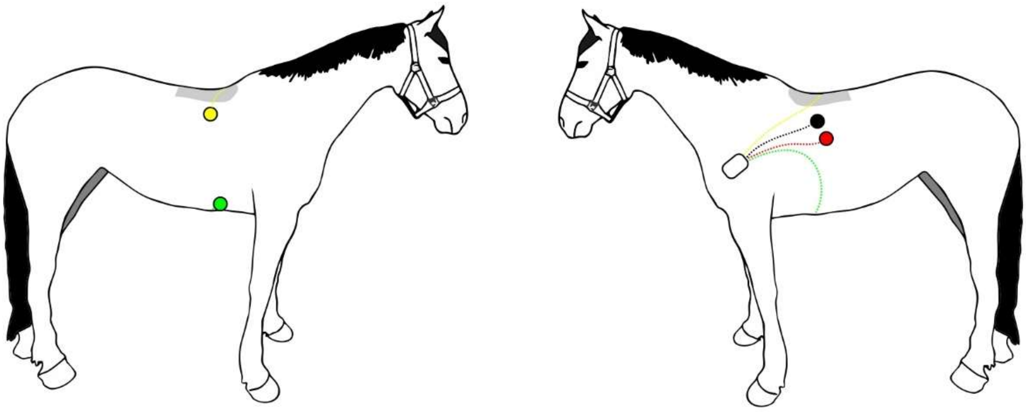

**Figure S2** Heart rate alterations during the exercise session. Red lines show the locations of automatically extracted 60s ECG strips.

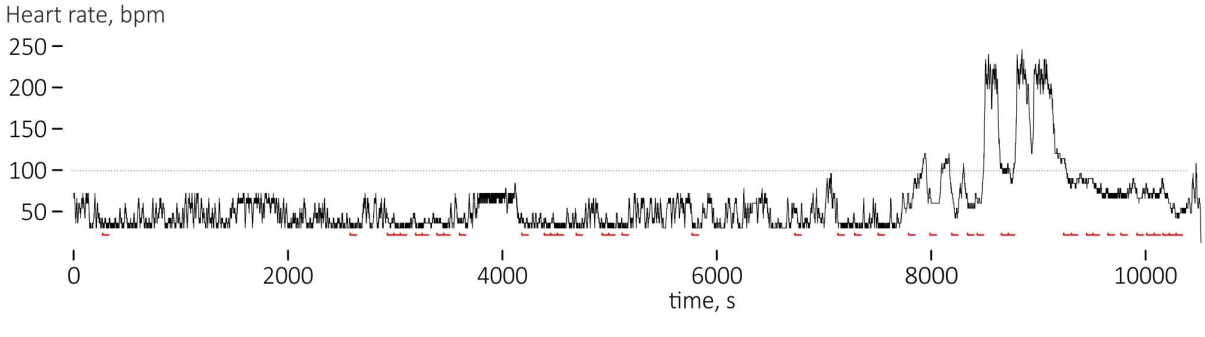

Supplement: Supplementary file 1 — Supplementary Information. [file 41598_2026_41281_MOESM1_ESM.pdf]
